# Trophic specialization and morphological divergence between two sympatric species in Lake Catemaco, Mexico

**DOI:** 10.1002/ece3.4042

**Published:** 2018-04-19

**Authors:** Claudia Patricia Ornelas‐García, Fernando Córdova‐Tapia, Luis Zambrano, María Pamela Bermúdez‐González, Norman Mercado‐Silva, Berenit Mendoza‐Garfias, Amando Bautista

**Affiliations:** ^1^ Departamento de Zoología Instituto de Biología Universidad Autónoma de México Mexico City Mexico; ^2^ Centro de Investigación en Biodiversidad y Conservación Universidad Autónoma del Estado de Morelos Cuernavaca Morelos Mexico; ^3^ Programa Institucional de Doctorado Facultad de Ciencias Naturales Campus UAQ‐ Juriquilla Universidad Autónoma de Querétaro (UAQ) Querétaro Mexico; ^4^ Centro Tlaxcala de Biología de la Conducta Universidad Autónoma de Tlaxcala Tlaxcala de Xicohténcatl Tlaxcala Mexico

**Keywords:** *Astyanax*, characids, diet, ecomorphology, lacustrine diversification, stable isotopes

## Abstract

The association of morphological divergence with ecological segregation among closely related species could be considered as a signal of divergent selection in ecological speciation processes. Environmental signals such as diet can trigger phenotypic evolution, making polymorphic species valuable systems for studying the evolution of trophic‐related traits. The main goal of this study was to analyze the association between morphological differences in trophic‐related traits and ecological divergence in two sympatric species, *Astyanax aeneus* and *A. caballeroi,* inhabiting Lake Catemaco, Mexico. The trophic differences of a total of 70 individuals (35 *A. aeneus* and 35 *A. caballeroi*) were examined using stable isotopes and gut content analysis; a subset of the sample was used to characterize six trophic and six ecomorphological variables. In our results, we recovered significant differences between both species in the values of stable isotopes, with higher values of δ^15^N for *A. caballeroi* than for *A. aeneus*. Gut content results were consistent with the stable isotope data, with a higher proportion of invertebrates in *A. caballeroi* (a consumption of invertebrates ten times higher than that of *A. aeneus*, which in turn consumed three times more vegetal material than *A. caballeroi*). Finally, we found significant relationship between ecomorphology and stable isotopes (*r* = .24, *p* < .01), hence, head length, preorbital length, eye diameter, and δ^15^N were all positively correlated; these characteristics correspond to *A. caballeroi*. While longer gut and gill rakers, deeper bodies, and vegetal material consumption were positively correlated and corresponded to *A. aeneus*. Our results are consistent with the hypothesis that morphological divergence in trophic‐related traits could be associated with niche partitioning, allowing the coexistence of closely related species and reducing interspecific competition.

## 1 INTRODUCTION

Understanding the mechanisms that influence speciation and their relationship with the appearance of new traits is a key question for evolutionary biologists. According to ecological speciation theory, adaptive divergence plays a key role in the formation and perpetuation of barriers between species that inhabit different environments (Schluter, 2001). In this scenario, the evolution of morphological traits could be influenced by adaptive processes, especially in the case of traits subject to divergent selection (Schluter, 2001). Thus, it is important to identify the main triggering elements of diversification related to phenotypic divergence. In this respect, there is evidence that ecological opportunities and competition might promote divergence between closely related species (Losos, 2010; Meyer, 1990; Sidlauskas, 2008), that is, trophic partitioning could trigger morphological changes associated with resource exploitation, which in turn promote speciation (Barluenga, Stölting, Salzburger, Muschick, & Meyer, 2006; Burress, Holcomb, & Armbruster, 2016; Gray & McKinnon, 2007). With respect to ecological traits, such as trophic‐related ones, polymorphic species provide a unique opportunity to assess the relationship between niche partitioning and morphospace evolution (Arbour & López‐Fernández, 2014; Magalhaes, Ornelas‐Garcıa, Leal‐Cardin, Ramírez, & Barluenga, 2015; Meyer, 1990).

In teleosts, there are several examples of phenotypic traits showing functional disparity (e.g., bimodal distributions) under ecological divergence, including the pharyngeal jaw (Barluenga et al., 2006; Burress et al., 2016; Hulsey et al., 2006; Magalhaes et al., 2015), the gill rakers (Rundle, Vamosi, & Schluter, 2003; Schluter & McPhail, 1992), body shape (Barluenga et al., 2006), and gut length (Wagner, McIntyre, Buels, Gilbert, & Michel, 2009). Together with other characteristics, such as mouth position (Bonato, Burress, & Fialho, 2017; Gerking, 1994), snout length (Bonato et al., 2017), fin length, body size (Farré, Tuset, Maynou, Recasens, & Lombarte, 2013), and locomotion mode (Webb, 1984), these traits provide evidence of the functional role played by a species in an ecosystem (Córdova‐Tapia & Zambrano, 2016). Hence, morphological characterization provides a good approximation of the feeding modes or prey types that are used differentially by polymorphic species (Barluenga et al., 2006; Bonato et al., 2017; Meyer, 1990; Wainwright, 1987, 1988).

In this regard, the genus *Astyanax* (Characidae) provides an excellent model system to study ecomorphological diversification (Mise, Fugi, Pagotto, & Goulart, 2013; Ornelas‐García, Bautista, Herder, & Doadrio, 2017; Santos, Camilo, Albieri, & Araújo, 2011), which is characterized by presenting a high morphological divergence in response to both biotic and abiotic conditions (Ornelas‐García, Domínguez‐Domínguez, & Doadrio, 2008). In our study, we considered two closely related species: *Astyanax aeneus* and *A. caballeroi,* that coexist in the tropical Lake Catemaco in Mexico, with conspicuous morphological differences mainly related to the teeth shape, dental formula, eye size, snout length, body depth, head profile, and mouth orientation (Contreras‐Balderas & Rivera‐Teillery, 1983; Ornelas‐García, Bastir, & Doadrio, 2014). These morphological differences between the two species have been suggested to correspond to alternative trophic niches (Miller, Minckley, & Norris, 2005).

In addition to morphological differences, we have proposed that functional modularity plays a role in the body and skull variation between the two species, probably in association with divergent evolution (Ornelas‐García et al., 2017). Modular evolution was observed in the preorbital region of the skull of *A. caballeroi*. In contrast, body shape variation of both species is partitioned into two modules, the head and the rest of the body, affecting both trophic morphology and swimming performance of the fish (Ornelas‐García et al., 2017; Webb, 1984).

Despite the high degree of morphological differentiation between the two species inhabiting Lake Catemaco, there is no evidence of trophic segregation between them. Therefore, the main goal of the present work was to assess the relationship between morphological disparity and trophic divergence between two sympatric species, *Astyanax aeneus* and *A. caballeroi*. To this end, 12 ecomorphological variables were evaluated between the two species, while trophic divergence was assessed by stable isotopes and gut content analyses. We expected to observe an association between ecomorphological variation and dietary habits, which would suggest a divergence in traits that are ecologically relevant for niche partitioning among sympatric characid species.

## 2 MATERIALS AND METHODS

### 2.1 Sample collection and taxonomic determination

**Figure 1:**
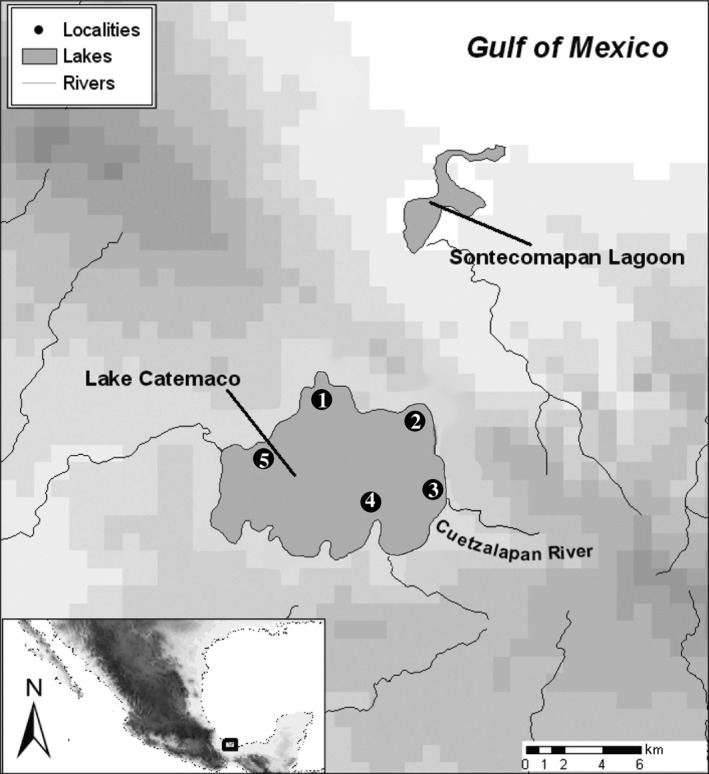
Map of the sampled localities in Lake Catemaco. **(**1) Agatepec Island, **(**2) Coyame, **(**3) Tebanca, **(**4) Margarita and **(**5) Chagos Island

A total of five localities in Lake Catemaco (located at Sierra de Los Tuxtlas, Veracruz, Mexico) were sampled; the sites were selected to include the different habitats present in the lake in terms of depth and substrate (Figure 1). The sampling was carried out between November 2012 and August 2013, during the dry and wet seasons. The fish were collected using two 30‐m long and 1.5‐m‐high gill nets of 12 different mesh sizes: 5, 6.25, 8,10, 12.5, 15.5, 19.5,24, 29, 35, 43, and 55 mm. After sampling, the fish were kept alive in aerated coolers until tissue processing (normally <1 hr after the last sampling). Under more sterile conditions, the fish were euthanized by putting them in ice water (around 4°C), and the stomach and muscle biopsies were collected. All processed specimens were preserved as vouchers in 95% ethanol and deposited in the National Fish Collection, IBUNAM. The fish were assigned to nominal species based on diagnostic morphological characters (Contreras‐Balderas & Rivera‐Teillery, 1983; Miller et al., 2005).

### 2.2 Stable isotopes and gut content analyses

#### 2.2.1 Stable isotopes

A total of 70 individuals (35 *Astyanax aeneus* and 35 *Astyanax caballeroi*) from Lake Catemaco were examined using stable isotope analysis (Table S1). Muscle tissue samples were collected individually, dried using silica gel and then frozen at −80°C. Samples were dried at 60°C for 48 hrs, ground into a fine powder with a mortar and pestle, and stored in sterile 2‐ml tubes. Subsamples of each ground sample were weighed, packaged into tin capsules and sent to the Center of Stable Isotopes at the University of New Mexico (Department of Earth and Planetary Sciences) to measure the stable isotope ratios of carbon (^13^C/^12^C) and nitrogen (^15^N/^14^N). The isotopic ratios were expressed in standard delta “δ” notation. The mean standard deviation between replicates was 0.04 ‰ for δ^13^C and 0.07 ‰ for δ^15^N.

The analysis of the trophic niche of both species was performed by adapting the community niche analysis using δ^13^C‐ δ^15^N bi‐plots (Layman, Arrington, Montaña, & Post, 2007; Zambrano, Valiente, & Vander Zanden, 2010). Trophic niche analysis evaluates the trophic niche of a single species based on the carbon range (CR), nitrogen range (NR), total niche area (TA), mean distance to centroid (CD), mean nearest neighbor distance (NND), and standard deviation of nearest neighbor distance (SDNND) (Layman et al., 2007). These parameters define the range of food sources consumed by certain species, the capacity of a species to consume organisms from different trophic levels, the trophic niche occupied by each species, and the similarity of food sources among individuals. Student's *t*‐test was performed to compare the mean differences between species for each stable isotope ratio; a multivariate analysis of variance (MANOVA) was performed when both isotopes were considered. Differences in carbon and nitrogen between seasons and species were analyzed using an analysis of variance (ANOVA).

#### 2.2.2 Stomach content

Only the stomach sack was used to compare the stomach content, and the intestines were discarded. Individuals with empty stomachs were omitted from the analysis. Dietary items were categorized into the following groups: detritus, vegetal material, invertebrates, and fish remains (eggs, eyes, scales, bones, teeth, fin rays, gills and fish vertebrae) (Table S2). We used these categories to estimate diet composition, using a standard gravimetric method to account for broad differences in diet habits (Zacharia & Abdurahiman, 2004). The wet weight of the food items was measured after removing superfluous water. The mean proportions of diet items were compared between species using a chi‐square test.

### 2.3 Morphology and trophic habits

Morphological and trophic data were correlated to characterize the ecomorphological patterns of the two sympatric species. Multivariate ordination analysis was carried out with dietary, isotopic, and morphological data for a total of 33 individuals (Table S3 and S4), with a similar proportion of each species (*A. aeneus*,* N* = 17; *A. caballeroi*,* N* = 16). A total of 12 measurements were included; of these, six were morphological, mainly related to locomotion and food acquisition; the other six corresponded to trophic‐related variables (Córdova‐Tapia, Hernández‐Marroquín & Zambrano, 2017; Montaña & Winemiller, 2013; Winemiller, 1991). The morphological variables included the head length (HL), standard length (SL), eye diameter (ED/(HL/SL), the preorbital region (PrOL/HL); the body depth (BD/SL); the gill raker length (GRL, i.e., the longest gill raker/SL); and the gut length (GL, i.e., intestine length/SL) (Table S4 and S5). Trophic data corresponded to the stomach content (fish remains, vegetal material, detritus and invertebrates) and stable isotope data (δ^13^C and δ^15^N). To equally weight each trait, the data were standardized using a z‐transformation; thus, the mean of each trait was equal to 0, and its standard deviation was equal to 1 (Córdova‐Tapia et al., 2017). Student's *t*‐test was performed to compare the mean differences between species for each morphological and trophic variable; a multivariate analysis of variance (MANOVA) was performed when the six variables were considered together.

Correspondence tests were performed, (1) between morphology (HL, ED, PROL, BD, GRL, and GL) and gut content (fish remains, vegetal material, detritus, and invertebrates), and (2) between morphology and stable isotopes signatures (δ^13^C and δ^15^N), using Mantel's test on the Euclidean distances between individuals. In addition, Spearman's correlation coefficients were estimated for each variable. Based on the correlation matrix of the 12 transformed variables, a principal component analysis (PCA) was carried out to compare the distribution of individual specimens. These analyses were performed in Past v.3 (Hammer, Harper, & Ryan, 2001).

## 3 RESULTS

### 3.1 Stable isotopes and gut content analyses

**Figure 2:**
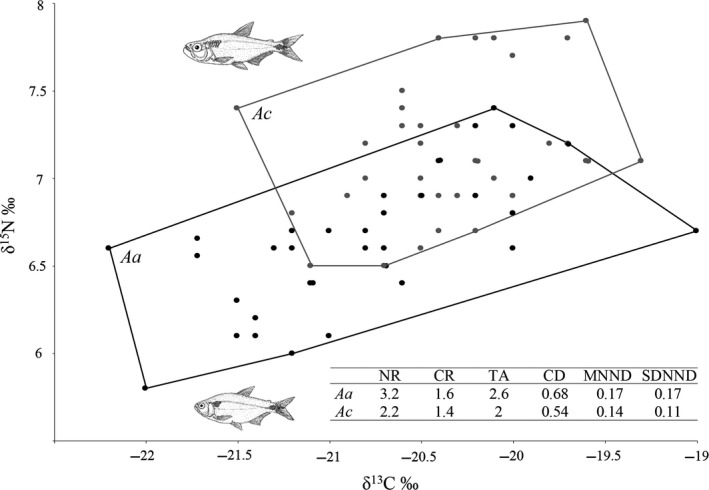
Trophic niche analysis of *A. aeneus* (black circles) and *A. caballeroi* (gray circles). Polygons encompass the convex hull area of all individuals. CD **,** mean distance to centroid; CR **,** carbon range; NR **,** nitrogen range**;** NND **,** mean nearest neighbor distance; SDNND **,** standard deviation of nearest neighbor distance**;** TA **,** total niche area

The bi‐plot comparing trophic niche areas revealed a substantial overlap in niche space between the two species in the middle of the trophic structure (Figure 2). However, significant differences were found between both species with respect to carbon (δ^13^C: *t* (68) = −3.27, *p* < .01) and nitrogen (δ^15^N: *t* (68) = −5.73, *p* < .001) values, as well as when both isotopes were considered together (δ^13^C + δ^15^N: *F* (2, 67) = 16.22, *p* < .001). *A. caballeroi* had a higher mean nitrogen value (δ^15^N: 7.2 ± 0.38) than *A. aeneus* (δ^15^N: 6.6 ± 0.39). The trophic niche analysis revealed that *A. aeneus* had higher NR, CR, TA, CD, NND, and SDNND than *A. caballeroi* (Figure 2). Additionally, *A. caballeroi* showed differences between seasons in δ15N (*F* (43) = 9.8, *p* < .01), but not in δ13C (*F* (46) = 0.13, *p* = .72). *A. aeneus* showed differences between seasons in δ13C (*F* (35) = 3.81, *p* = .05) and δ15N (*F* (34) = 6.3, *p* = .01). The comparison between both species showed significant differences only during the wet season in both δ13C (*F* (57) = 9 *p* < .01) and δ15N (*F* (53) = 10.68, *p* < .01).

**Figure 3:**
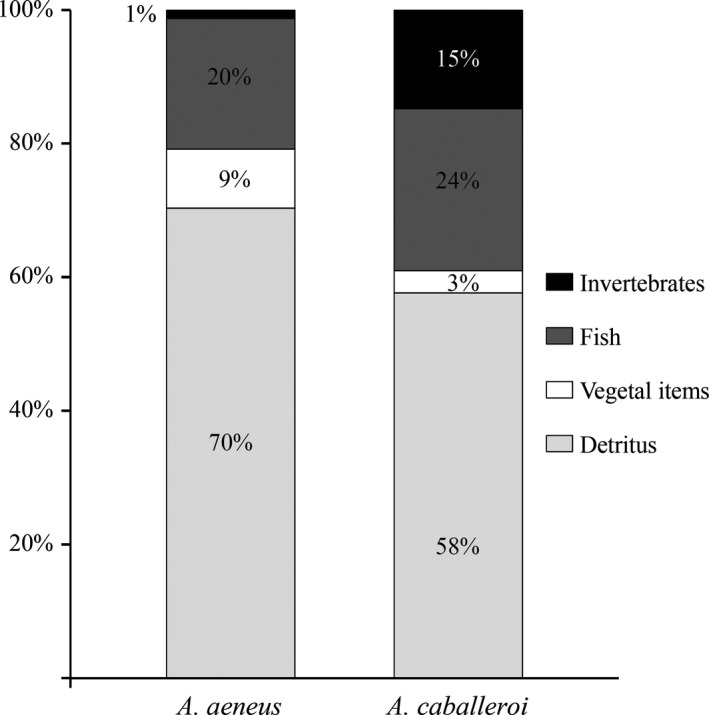
Relative compositions (biomass) of gut contents of *A. aeneus* and *A. caballeroi*

Regarding the stomach content analysis, detritus was the dominant item in both species (Figure 3), although significant differences were found in the mean proportions of diet items (χ^2^ = 16.74 (3), *p* < .001). *A. caballeroi* showed a higher consumption of invertebrates (14.8%) and fish debris (24.2%) than *A. aeneus*, which showed values of 1.29% and 19.55%, respectively. Furthermore, *A. aeneus* had a higher proportion of vegetal items than *A. caballeroi* (8.8% vs. 3.3%, respectively, Table S2).

### 3.2 Morphology and trophic habits

**Figure 4:**
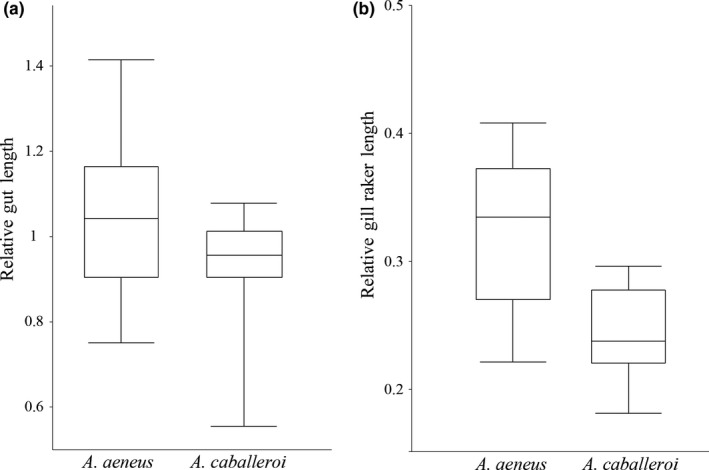
Boxplots of the comparisons of relative gut length and relative gill raker length between *A. aeneus* and *A. caballeroi*. Both relative gut length and gill raker length were significantly different (*p* < .05)

There were differences between both species in the GRL and the GL (Figure 4). The GRL of *A. aeneus* was longer than that of *A. caballeroi*; that is, GRL_MEAN_ 0.32 ± 0.05 vs GRLMEAN 0.24 ± 0.03, respectively (*t* (32) = 5.10, *p* < .01, Figure 4). Similarly, *A. aeneus* had a significantly longer GL than *A. caballeroi;* that is, GL_MEAN_ 1.04 ± 0.17 vs GL_MEAN_ 0.89 ± 0.18, respectively (*t* (32) = 2.26, *p* < .05). Significant differences between species were also found in HL (*t* (32) = −4.73, *p* < .01) and ED (*t* (32) = −4.58, *p* < .01), while nonsignificant differences were found with respect to PROL (*t* (32) = 0.52, *p* = .6) and BD (*t* (32) = 1.46, *p* = .15). When all morphological variables were considered, significant differences were found between both species (*F* (6, 26) = 6.28, *p* < .01).

Mantel's test showed a significant relationship between morphology and stable isotopes (*r* = .24, *p* < .01). However, no significant correlation was found between gut content and morphology (*r* = −.11, *p* = .88), nor between gut content and stable isotopes (*r* = −.12, *p* = .89). Spearman's correlation coefficients between variables (Table 1) showed a negative and significant correlation between HL and the next variables: BD, GRL, and GL. Moreover, there was a positive and significant correlation between the next pairs of variables: HL and ED; δ^15^N and δ^13^C; ED and δ^15^N; and GRL and GL. In this respect, *A. caballeroi* had a longer HL, a slender body (BD), shorter GRL, shorter GL, larger ED, and higher ratios of δ^15^N and δ^13^C. In contrast, the individuals of *A. aeneus* showed a deeper body (BD), longer gut length (GL) and gill rakers, and lower values of δ^15^N.

**Table 1:** Spearman's correlation among gut content, morphology, trophic traits, and stable isotopes

	*p* – value											
	Gut content				Morphology				Trophic traits		Stable isotopes	
	FSH	VM	D	INV	HL	ED	PROL	BD	GRL	GL	δ15N	δ13C
Spearman's *r*s												
Gut content												
FSH		**.05**	.19	.42	.84	.71	.21	.37	.88	.26	.47	.13
VM	−**.35**		.34	.95	.49	.94	.06	.13	.91	.93	.35	.53
D	−.67	−.17		.09	.36	.21	.87	.61	.47	.14	.74	.01
INV	−.14	.01	−.29		.19	.68	.41	.71	.49	.29	.58	.95
Morphology												
HL	.03	−.12	−.16	.23		**<.01**	**<.01**	**.01**	**<.01**	**<.01**	**<.01**	**<.01**
ED	.07	.01	−.22	−.07	**.48**		.86	.88	.15	**.02**	**<.01**	.17
PROL	−.22	.31	−.02	.14	−.28	−.03		.12	.34	.63	.66	.29
BD	−.16	.26	.09	.06	−**.42**	.02	.27		.09	.38	.44	**<.01**
Trophic traits												
GRL	−.02	.01	.12	.12	−**.73**	−**.67**	.16	.29		**<.01**	**<.01**	**.01**
GL	−.21	.01	.26	.18	−**.52**	−**.72**	.08	.15	**.83**		**.04**	.06
Stable isotopes												
δ15N	−.13	.16	−.05	.09	**.49**	**.46**	.07	−.14	−**.51**	−**.37**		**<.01**
δ13C	.26	.11	−.41	−.01	**.58**	.24	−.18	−**.48**	−**.43**	−.31	**.63**	

FSH: fish remains; VM: vegetal material; D: detritus; INV: Invertebrates; HL: head length; ED: eye diameter; PrOL: pre‐orbital length; BD: body depth; GRL: relative gill raker length; GL: relative gut length. The significant correlation P values are in bold.

**Figure 5:**
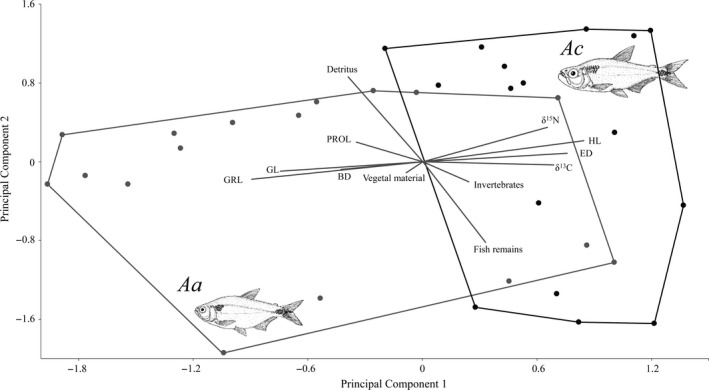
Principal component analysis considering values related to gut content, morphology, trophic traits, and stable isotopes values. BD, body depth; ED, eye diameter; HL, head length; PrOL, pre‐orbital length; RGRL, relative gill raker length; RGL, relative gut length Aa= *Astyanax aeneus* and Ac= *Astyanax caballeroi*

The PCA results allowed us to explore morphospace differences between both species; the first and second components accounted for 50.6% of the total variance (36.1% for PC1 and 14.5% for PC2; Table 2, Figure 5). The positive scores of PC1 were associated with HL, ED, δ^15^N, δ^13^C, invertebrates, and fish remains, while the negative scores were associated with GRL, GL, BD, and PrOL. PC1 indicated a greater segregation between the two species than PC2. Specifically, the positive components of PC1 identified the *A. caballeroi* individuals with shorter GL, GRL, higher consumption of invertebrates and fish remains, and higher values of δ^15^N; in contrast, on the negative side of the PC1, we found *A. aeneus* individuals with shorter PrOL, deeper BD, and higher consumption of vegetal material and detritus. PC2 ordering included detritus on the positive side and fish remains on the negative side (Table 2, Figure 5).

**Table 2:** Results of the principal component analysis for values related to gut content, morphology, trophic traits, and stable isotopes values. For each principal component, the highest correlations (≥60) are highlighted in bold

	PC 1	PC 2
Eigenvalue	4.2	1.74
% variance	36.1	14.5
% cumulative	36.1	50.6
Gut content		
Fish remains	0.33	−**0.82**
Vegetal material	−0.09	−0.12
Detritus	−0.41	**0.86**
Invertebrates	0.25	−0.21
Morphology		
HL	**0.85**	0.21
ED	**0.76**	0.08
PROL	−0.35	0.20
BD	−0.43	−0.08
Trophic traits		
GRL	−**0.89**	−0.18
GL	−**0.75**	−0.09
Stable isotopes		
δ^15^N	**0.65**	0.35
δ^13^C	**0.69**	−0.03

BD, body depth; ED, eye diameter; GRL, gill raker length; GL, gut length; HL, head length; PROL, pre‐orbital length.

## 4 DISCUSSION

Ecological opportunity has been proposed as a key element in the colonization of new habitats, and it is believed to contribute to morphological diversification by providing a means to exploit alternative resources (Losos, 2010). The evolution of a trait that enables the exploitation of a new resource has a strong influence in the diversification of species as well as in shifts of trophic and habitat niches (Burress et al., 2016; Losos, 2010). Thus, morphology has been considered a good indicator of ecology and feeding habits (Gatz, 1979).

In characiforms, previous studies have found correlations between ecomorphological traits and trophic ecology, which can help us to understand the factors that enable the coexistence of closely related species (Bonato et al., 2017; Mise et al., 2013). The present study corroborated the occurrence of a significant correlation between ecomorphological traits and trophic habits (i.e., stable isotopes values) of two sympatric species of *Astyanax* genus. As such, these results provide additional evidence supporting the hypothesis that morphology could reflect the ecology of an organism and could help predict its trophic habits (Bonato et al., 2017).

### 4.1 Stable isotopes and gut content analyses

In the present study, a significant correlation between stable isotopes and ecomophology was observed, in contrast to the latter and diet content. In this respect, diet analysis provides a snapshot of fish feeding habits and shows temporal variations that could be difficult to approach. Stable isotopes analysis provides a time‐integrated indicator of energy resources (Vander Zanden, Casselman, & Rasmussen, 1999). When combined, these two sources of evidence provide robust analytic tools that can be used to better understand the consumption and assimilation of food by fish (Vander Zanden & Vadeboncoeur, 2002). Stable carbon and nitrogen isotope ratios have been widely used to provide a time‐integrated perspective of feeding relationships (Vander Zanden, Casselman, et al., 1999). The nitrogen stable isotope signature (δ^15^N) increases proportionally to the trophic position in the food web and shows habitat‐dependent variation; in contrast, a source of variation in the carbon stable isotope (δ^13^C) could be associated with the base of the food webs among other factors (Vander Zanden, Shuter, Lester, & Rasmussen, 1999; Vander Zanden, Casselman, et al., 1999).

This study confirmed that *Astyanax* species are an opportunistic group (Mise et al., 2013) with the ability to exploit a wide variety of trophic resources; however, some important differences were found between the two coexisting species studied here. For example, δ^15^N and δ^13^C showed wider variation in *A. aeneus* (Figure 2), which has been suggested as a characteristic of fish with omnivorous habits in lacustrine systems (Córdova‐Tapia, Contreras, & Zambrano, 2015; Jepsen & Winemiller, 2002; Vander Zanden & Vadeboncoeur, 2002). In this respect, the niche area of *A. caballeroi* was more restricted than that of *A. aeneus* (TA = 2 and 2.6, respectively), which is characteristic of fish with specialized trophic habits (Jepsen & Winemiller, 2002). The results of our stomach content analysis showed that this interpretation coincides with the trophic habits of *A. caballeroi*, which showed a much higher consumption of invertebrates than *A. aeneus*, which in turn showed a higher consumption of vegetal material.

### 4.2 Morphology and trophic habits

Our results showed that the feeding patterns were correlated with morphological differentiation. Firstly, we observed bimodal distributions of trophic‐related traits between species, that is, *A. aeneus* showed longer gut length and gill raker than did *A. caballeroi*. Additionally, a significant correlation between isotopic signatures and morphology was recovered by Mantel's test. Thus, although *Astyanax* species are considered omnivorous and opportunistic species, our results show their capability to diverge in terms of eco‐morphology, reducing interspecific competition, as has been shown in other sympatric *Astyanax* species (Mise et al., 2013; Santos et al., 2011). *A. caballeroi* showed a comparatively higher δ^15^N, larger head, slender body, shorter gut length, and shorter gill rakers, as well as a 10‐fold higher consumption of invertebrates than *A. aeneus*. The combination of this type of head morphology and an elongated body could be associated with predatory habits (Arbour & López‐Fernández, 2014). Other studies have shown that longer lower jaws, upturned mouths, and greater snout lengths are correlated with carnivorous diets (Bonato et al., 2017; Burress et al., 2016; Cooper, Wernle, Mann, & Albertson, 2011), as has been reported for the neotropical characid genus *Oligosarcus* (Bonato et al., 2017; Santos et al., 2011).

In contrast, *A. aeneus* showed a deeper body, longer gill rakers, longer gut length, and a higher proportion of vegetal material in the stomach content, while its stable isotope signatures were consistent with those of an omnivorous species (Jepsen & Winemiller, 2002; Vander Zanden & Vadeboncoeur, 2002). Other omnivorous species of *Astyanax* in South America that consume large amounts of detritus and vegetation show similar morphological patterns, that is, deep bodies, longer intestines, and small mouths (Bonato et al., 2017). Furthermore, short oral jaws have been associated with a powerful bite and are considered advantageous for low‐mobility or immobile food items (Arbour & López‐Fernández, 2014; Cooper et al., 2010; Wainwright & Richard, 1995).

Based on both ecomorphology and diet, we can provide some suggestions regarding prey capture behavior for both species. *A. caballeroi* depicts a large head, upwardly facing mouth position, fusiform body, and conical teeth, consistent with the capture of elusive preys and access to floating terrestrial insects (e.g., hymenopterans or coleopterans) (Bonato et al., 2017). In contrast, *A. aeneus* had a relatively deeper body and shorter snout, which is associated with the ability to move upward and downward in the water column, from the bottom to the surface, as befits an omnivorous species (Gatz, 1979; Mise et al., 2013; Winemiller, 1991).

In conclusion, the *Astyanax aeneus* and *A. caballeroi* model system showed a significant correlation between ecomorphological traits and trophic habits (i.e., stable isotopes values), supporting the hypothesis that morphological divergence in trophic‐related traits is associated with niche partitioning. This study also provides additional evidence that trophic partitioning could trigger morphological changes and ultimately promote speciation, as seen in other groups of fishes.

## CONFLICT OF INTEREST

None declared.

## AUTHOR CONTRIBUTIONS

CPOG conceived and designed the experiments. PBG, BGM, CPOG, and FC performed the experiments. CPOG and FC analyzed the data. CPOG, LZ, AB, and NMS contributed reagents/materials/analysis tools. CPOG, FC, LZ, PBG, NMS, BMG, and AB wrote the manuscript. FC, CPOG, and PBG prepared the figures and/or tables. CPOG, BGM, LZ, AB, FC, NM, and PBG reviewed drafts of the manuscript.
