# Supplementary figures and images for: Trophic specialization and morphological divergence between two sympatric species in Lake Catemaco, Mexico
Source: Ecol Evol. 2018 Apr 19;8(10):4867–75. doi: 10.1002/ece3.4042 (PMC5980260; doi:10.1002/ece3.4042)

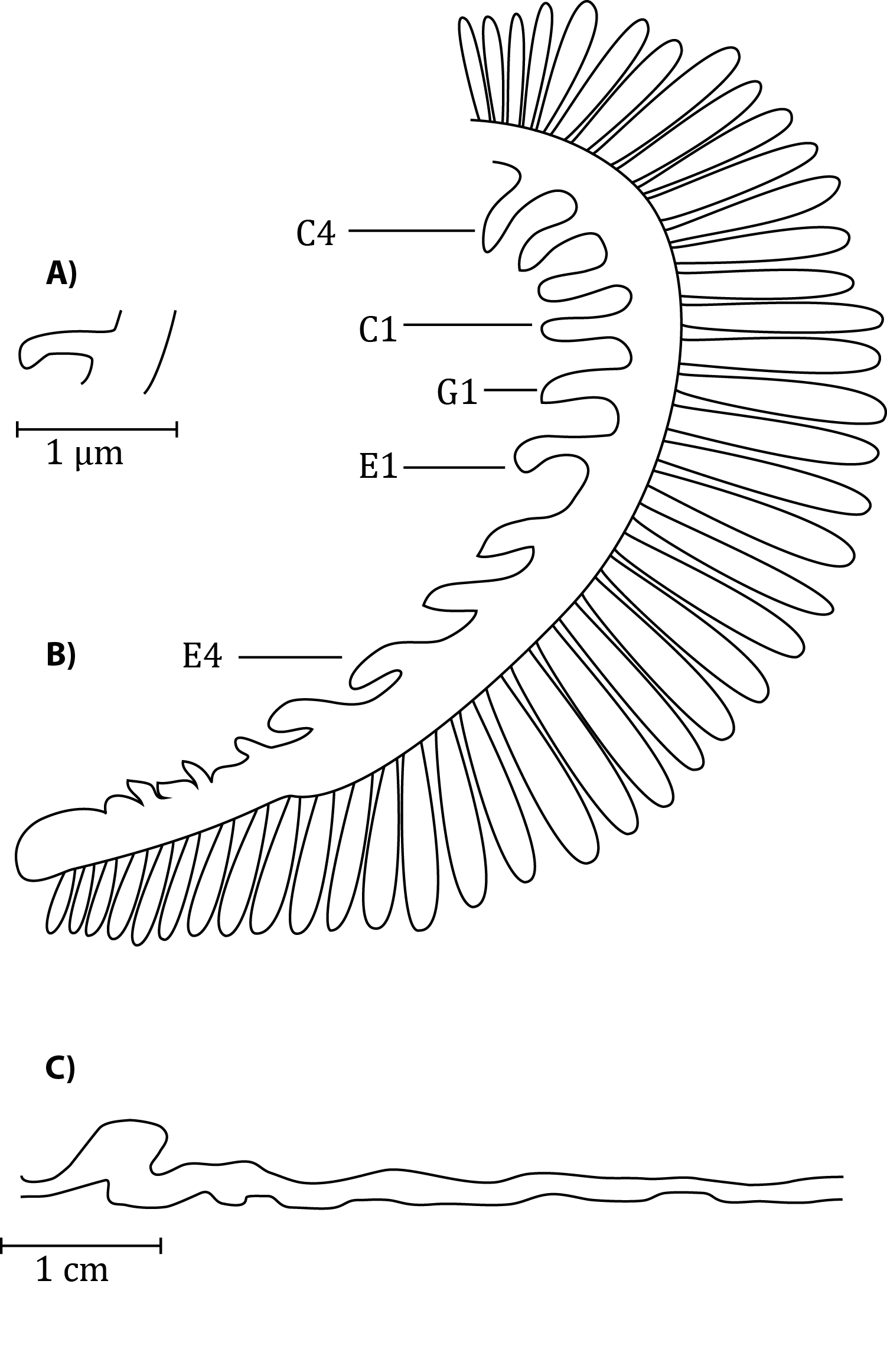

Supplement: Supplementary file 2 [file ECE3-8-4867-s002.tif]
